# Supplementary material for: Trauma clinicians’ views of physical exercise as part of PTSD and complex PTSD treatment: A qualitative study
Source: PLOS Ment Health. 2024 Sep 3;1(4):e0000114. doi: 10.1371/journal.pmen.0000114 (PMC12798193; doi:10.1371/journal.pmen.0000114)
Supplement: S1 Text — (DOCX) [file pmen.0000114.s001.docx]

**Interview Guide**

**Initial question:**
1. Tell me about your clinical work here.
- What are the main/chief difficulties experienced by your patients?

- What kinds of treatments do you offer?

**Main Questions:**

2. What do you think about recommending physical exercise as an adjunctive (supportive/additional) intervention for PTSD/CPTSD?

Prompts:

- What kind of physical exercises would you recommend?

- Can you give me an example of someone you have worked with where you have encouraged them to use physical exercise, or thought it might be helpful?

- How did you decide on recommending it to them?

- How did you approach bringing physical exercise up to your client? What was their response?

3. What do you think are the barriers to delivering exercise as an adjunctive treatment for PTSD/CPTSD?
- How do you think any of these could be overcome?

4. What do you think could facilitate the use of physical exercise as an adjunctive treatment for PTSD/CPTSD?
- How do you think we could better include Physical exercise in the multidisciplinary management of PTSD/CPTSD?

5. Is there something which you would like to add which I did not ask?
