# Supplementary material for: Trauma clinicians’ views of physical exercise as part of PTSD and complex PTSD treatment: A qualitative study
Source: PLOS Ment Health. 2024 Sep 3;1(4):e0000114. doi: 10.1371/journal.pmen.0000114 (PMC12798193; doi:10.1371/journal.pmen.0000114)
Supplement: Attachment — Submitted filename: Response to Reviewers May 2024 FINAL.docx [file pmen.0000114.s002.docx]

UCL Division of Psychiatry

**LONDON’S GLOBAL UNIVERSITY**

6^th^ Floor, Maple House

149 Tottenham Court Road

London, UK, W1T 7NF

PLOS Mental Health

29^th^ April, 2024

Dear Editor and Reviewers

Thank you for reviewing our research paper “Trauma clinicians' views of physical exercise as part of PTSD and CPTSD treatment: A qualitative study”. We are grateful for your feedback and the opportunity to strengthen the paper.

We have addressed each of your points in turn in the table below.

| Comments from Editor | | |
| --- | --- | --- |
| We ask you to expand the abstract for clarity on identified barriers and benefits, provide comprehensive details on study design, methodology, and data analysis. It's essential to deepen the discussion around the role of trauma clinicians, compare barriers to those in other mental disorders, and describe a trauma-informed exercise environment in detail. Incorporating client feedback and discussing neurobiology's role in PTSD/CPTSD treatment will strengthen your manuscript. Please also correct any statistical and grammatical issues. Your revised submission should address these concerns in a consolidated manner for re-evaluation. | Thank you for this feedback. We have addressed these points, in line with the reviewers’ feedback, below.  Please note that statistical issues are not relevant to this paper as it is a qualitative study, not a quantitative study. |  |
| Comments from Reviewer #1 | | |
| The article is great, I enjoyed reading it :) The only thing is to expand a bit the Abstract, in the Result section, so it will be more clear from the beginning, what kind of barriers were identified, and what kind of benefits. | Thank you for your positive feedback.  We have expanded the Results sub-section of the Abstract to provide more detail about the benefits and barriers that were identified. | Abstract p1 |
| Comments from Reviewer #2 | | |
| Firstly, the sample is small. There is no mention of data saturation | We are aware that this is a relatively small sample of specialist clinicians, so have already acknowledged this (alongside other limitations with the sample) in the Strengths and Limitations section within the Discussion (page 19). Given the stated sample limitations, we have therefore been careful to only tentatively conclude that “this study provides some preliminary evidence…” (page 19).  Please note that “data saturation” is a concept associated with Grounded Theory, not Reflexive Thematic Analysis, and is now largely dismissed as a helpful concept in qualitative research. For a more detailed discussion of why this is, please see: Braun, V., & Clarke, V. (2021). To saturate or not to saturate? Questioning data saturation as a useful concept for thematic analysis and sample-size rationales. *Qualitative research in sport, exercise and health*, *13*(2), 201-216. | Discussion p24  Conclusion p25 |
| Secondly, the article would benefit from more depth re the role of these trauma clinicians in determining treatment programs. In other words, are these the group of professionals who decide treatment approaches for the client. | Thank you for this suggestion. In the UK, specialist trauma services are provided by expert specialist trauma clinicians, usually in tertiary mental health services. We have made this point clearer in the introduction and methods sections. | Introduction p5  Methods p5 |
| The barriers and facilitators outlined are as would be expected. It would benefit from a discussion of how these compare and contrast with those relevant to clients with other mental disorders. In other words, are individuals with PTSD or CPTSD unique in any way? | We have added some discussion of how the identified barriers and facilitators compare with clients with other mental health disorders in the Discussion section. | Discussion, p.19 |
| There is talk of a trauma informed exercise environment? What would this look like? There is talk about individuals within the gym being able to support the individual if they were triggered. What would this look like? Would this require training to be put out across gym staff? Is this feasible? It would benefit from more of an in-depth discussion of gym exercise versus outdoor activity given the symptom profile associated with PTSD and CPTSD. | Thank you for raising these questions. We have included some additional discussion of this in the Discussion section. | Discussion, p20 |
| I know that there is a discussion of client factors, but it would also benefit from evidence from clients. | We agree that this field of research will be enhanced by exploring the views of clients with PTSD and CPTSD, and we intend to continue this programme of research with a further study with service users in the future. We have added this point in the strengths and limitations section of the Discussion. | Discussion, p20 |
| Comments from Reviewer #3 | | |
| It is an interesting study that is giving an insight on the Trauma clinicians' views of physical exercise as part of PTSD and CPTSD treatment. All along in literature, Exercise has been documents for be crucial in maintaining body and brain health with few studies done to support this. So any study like this one is vital in advancing the knowledge in this area. | Thank you for your encouraging feedback. We agree that a holistic approach to physical and mental health is really important, and clinicians play a vital gatekeeping role in whether this is incorporated into treatment programmes or not. |  |
| Title • Avoid abbreviations in the title – Write “PTSD and CPTSD” in full | We have discussed this in research team and looked at previous studies on PTSD published in PLOS ONE and noted that most papers actually use ‘PTSD’ in their titles, rather than spelling out ‘post-traumatic stress disorder’. Given that PLOS Mental Health is a specific mental health journal, we felt that your readership would be familiar with the term PTSD, and that retaining this would keep the title of the paper shorter and easier to read. We did however agree that readers may be less familiar with the concept of ‘CPTSD’, so have changed the title to include ‘Complex PTSD’. We have already included the full terms and their abbreviations in the introduction. | Title p1 |
| Abstract • What was the study design? • How was the interviews conducted to collect the data? • Conclusion is not based on the key findings of the study | Thank you for these suggestions. We have expanded the Abstract to provide additional information on these aspects and changed the Conclusion section so it is closer to the key findings reported in the study. | Abstract p1 |
| Methods section • How were the specialist traumas clinicians identified and recruited in the study? How was the study participants selected? • In data collection, was the structured interviews conducted using a designed tool as a guide? Were responses to saturation able to be reached?? • The statement “Clinical contacts were also asked to circulate details of the study to other clinical colleagues in specialist trauma services----“What type of study design is this? The study design needs to be clearly explained!!!!! • What were the variables captured? • Which statistical package was used to summarize the data if any? • How was the ethical issues handled i.e. study approval and number if possible or waiver was given? | We have expanded the Methods section to provide additional detail about the aspects highlighted.   - We have added that we used purposive and snowballing techniques to recruit participants who were specialist trauma clinicians working in specialist tertiary trauma services across the UK. - The semi-structured interview guide was designed by the research team in line with the study’s research questions, as is usual in explorative qualitative research. We have already included information about the questions asked, and have included the interview guide in the Supplementary Information - Please see above comments about data saturation in the response to reviewer #2 and how this is not an approach consistent with reflexive thematic analysis. - The approach of asking contacts to circulate information about a study to other contacts is consistent with snowball sampling and is a frequently used method in qualitative research to access hard to reach groups. We have added clarification in the manuscript that we used a purposive and snowballing approach to recruitment. - There were not any variables captured, as this is not a quantitative study. - We did not use a statistical package to summarize the data as this is not a quantitative study. - Information about the study’s ethical approval and registration number is already provided in the sub-section on *Ethics* at the beginning of the Methods section. | Methods p5-7 |
| Results • Responses in table 1should be reported as % (n) or add another column for %. • Characteristic item on “Occupation”, there was no Psychiatrist!!! • 3.1. The Potential Benefits of Physical Exercise -All participants saw value in physical exercise. What were those values mentioned? • 3.1.2. Mind and Body: holistic treatment – same as above • 3.1.3. Reclaiming Life and the Body • Physical exercise was perceived as a tool for reclaiming goals around life and their bodies. Many forms of physical exercise were used in treatment – what were those different forms encountered? | We have added additional information in the Results section to address these points.   - We have added in %s for sample characteristics. - Most specialist tertiary level trauma services in the UK are provided by clinical psychologists, counselling psychologists and CBT therapists, in line with clinical guidance that psychological therapy is the first line intervention for PTSD and CPTSD. Psychiatrists very rarely work in specialist trauma services, and if they are doing so, it is usually as they are gaining/have additional psychotherapy qualifications. - Heading 3.1 is an introduction to the theme, and points 3.1.1 to 3.1.5 elaborate on the values perceived by the clinicians and how they perceived their potential mechanisms of effect. - We have provided some examples of the sorts of physical exercise which was recommended by clinicians. | Results p7-16 |
| Discussion • Discussion should also bring in the role of Neurobiology on Physical exercise and how it can be helpful in prevention of PTSD and CPTSD and how it can influence treatment of affected patients | We have returned to neurobiology and physical exercise benefits in the discussion, however, we have kept this brief, as we have already discussed this extensively in the Introduction section, and because this paper’s primary research questions is about *specialist trauma clinicians’ views about physical exercise and the barriers and facilitators they perceive in incorporating it into clients’ treatment, rather than about the neurobiological benefits of exercise per se*. |  |
| Conclusion • The key conclusion that can be drawn from the findings of the study are not well elaborated • Grammar and tenses need to be addressed – See 2nd sentence | We have revised the Conclusion section |  |
| Acknowledgments: Missing | We have taken the opportunity to add an Acknowledgements section to the manuscript. | Acknowledgements p20 |
| Journal Requirements | | |
| Please update your online Competing Interests statement. If you have no competing interests to declare, please state: “The authors have declared that no competing interests exist.” | We have updated this online. |  |
| Please ensure that the Title in your manuscript and the Title in your online submission form are the same. | We have checked this. |  |
| Please provide separate figure files in .tif or .eps format only and remove any figures embedded in your manuscript file. Please also ensure that all files are under our size limit of 10MB. You may leave the figure captions or legends in the manuscript. | We have deleted the figure from the main manuscript and have included it as a separate file. |  |
| We have noticed that you have uploaded Supporting Information files, but you have not included a list of legends. Please add a full list of legends for your Supporting Information files after the references list. | We have added a legend for the supporting information file. |  |

Thank you again for your time taken to review our paper. We hope these amendments address the points raised and very much look forward to hearing further from you.

With kind regards.

**Natasza Biernacka, Shivangi Talwar and Jo Billings**
