# Supplementary material for: Trauma clinicians’ views of physical exercise as part of PTSD and complex PTSD treatment: A qualitative study
Source: PLOS Ment Health. 2024 Sep 3;1(4):e0000114. doi: 10.1371/journal.pmen.0000114 (PMC12798193; doi:10.1371/journal.pmen.0000114)
Supplement: Attachment — Submitted filename: Response to Reviewers July 2024 FINAL.docx [file pmen.0000114.s003.docx]

UCL Division of Psychiatry

**LONDON’S GLOBAL UNIVERSITY**

6^th^ Floor, Maple House

149 Tottenham Court Road

London, UK, W1T 7NF

PLOS Mental Health

22^nd^ July, 2024

Dear Editor and Reviewer

Re: Trauma clinicians' views of physical exercise as part of PTSD and CPTSD treatment: A qualitative study.

Thank you for reviewing the amendments we made to our manuscript in light of your previous feedback, and providing a few further comments. We have responded to each of your points below.

| Journal Requirements | | |
| --- | --- | --- |
| Please review your reference list to ensure that it is complete and correct. If you have cited papers that have been retracted, please include the rationale for doing so in the manuscript text, or remove these references and replace them with relevant current references. Any changes to the reference list should be mentioned in the rebuttal letter that accompanies your revised manuscript. If you need to cite a retracted article, indicate the article’s retracted status in the References list and also include a citation and full reference for the retraction notice. | We have carefully reviewed the whole manuscript and reference list and revised these to make sure that all references are consistently reported and that all references are appropriately included in the reference list. | Throughout and References p22 |
| Comments from Reviewer #3 | | |
| Reviewer #3: Comments: Thanks for the revision and most of the comments have been addressed by the authors. However, some minor one below need to be addressed. | Thank you for this feedback. | - |
| 1. General comments and some key concerns: • Use past tense instead of future tense in sentence construction in the document • Limit use of first and second person in sentence construction. Instead use third person. | We have checked the manuscript and that findings are reported appropriately in the past tense. We have retained first person sentence construction where appropriate in the Methodology and Strengths & Limitations sections as this is encouraged in modern academic writing, particularly qualitative research, where the researchers are considered to be an integral part of the research. For example, see [First-person pronouns (apa.org)](https://apastyle.apa.org/style-grammar-guidelines/grammar/first-person-pronouns) | Checked throughout |
| 2. Methods section • Insert “Methodology” instead of “Methods” • Which statistical package was used to summarize the data in table 1? Is this data qualitative or quantitative? | We have changed “Methods” to “Methodology”.  We did not use a statistical package to summarise data in Table 1. Table 1 is a simple descriptive table reporting the socio-demographic characteristics of the 12 participants. This data is reported as simple frequencies and percentages were worked out with a calculator. We did not feel the need to explain this in the manuscript. | Methodology p5 |
| 4. Results • Responses in table 1- Insert “Responses (%, n)” as sub-title in the cell and then remove % on each of the figures • The % should come first i.e. 75.0 (9) and Write % to 1 decimal place all through the table | The data reported in Table 1 describe participant characteristics, rather than responses, therefore we have retained the heading “Number of Participants (%)”. As this is a qualitative study and the number of participants is small, we did not feel it was appropriate to place percentages before the number of participants in each category. We have retained the % after the number for clarity. We have provided percentages to 1 decimal place. | Results, p7-8 |

Thank you again for your time taken to review our paper. We hope these amendments address the points raised and very much look forward to hearing further from you.

With kind regards.

**Natasza Biernacka, Shivangi Talwar and Jo Billings**
